# Exploration of expanded carbohydrate chemical space to access biological activity using microwave-induced acid condensation of simple sugars†

**DOI:** 10.1039/d2ra01463g

**Published:** 2022-04-08

**Authors:** James Andrew London, Sarah Louise Taylor, Igor Barsukov, Alan Cartmell, Edwin Alexander Yates

**Affiliations:** Department of Biochemistry & Systems Biology, ISMIB, University of Liverpool Liverpool L69 7ZB UK eayates@liv.ac.uk

## Abstract

Complex glycans are ubiquitous in nature and essential to life. Despite their diverse roles, however, only a fraction of their potential chemical space has been explored. New regions of this chemical space can, nevertheless, be accessed by generating structures that do not occur in nature or by modifying naturally-occurring polysaccharide structures – collectively, termed new polysaccharides (NPs). Two synthetic routes to NPs are described; the *de novo* route, directly from monosaccharide starting materials and the functionalization route, involving glycosylation of existing polysaccharides. The reaction involves a simple condensation step under microwave heating, catalysed by environmentally benign organic acids and is illustrated by the generation of structures with biological activities ranging from cell signalling and inhibition of bacterial growth, to mimicking carbohydrate antigens of pathogenic microorganisms. The method is as applicable to fine chemicals as it is to industrial waste, for example, biotechnologically-derived d-allulose (d-psicose), or the waste products of biofermentation. Accessing this chemical space unlocks new functionalities, generating complex glycans with applications in the biological, medical, biotechnological and materials science arenas.

## Abstract 2

Condensation of simple sugars provides new polysaccharides with diverse biological activities, expanding access to carbohydrate chemical space.

## Graphical abstract

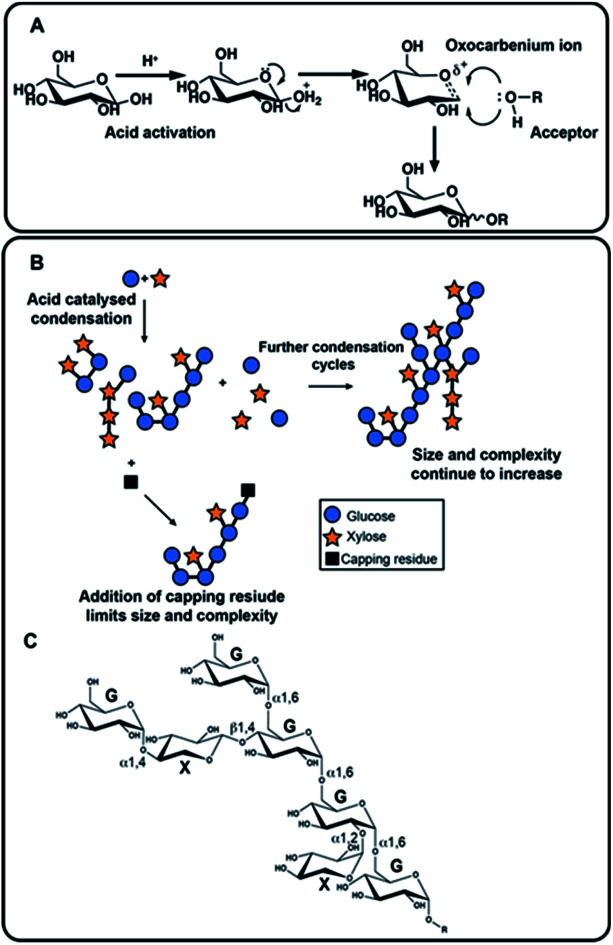

## 1. Introduction

Polysaccharides are some of the largest and most complex macromolecules as well as the most abundant. They perform essential biological roles in every kingdom of life. For instance, the extracellular matrix surrounding the cells of all mammals contains the complex glycan, heparan sulfate (HS), which has staggering potential complexity; an octasaccharide sequence offering thousands of hypothetical permutations, and its improper formation is embryonically lethal.^1^ The surfaces of many bacterial cells are also coated in glycans which form capsules known to protect the bacteria from viral entry and help defend their host against viral infection, whilst complex bacterial communities produce carbohydrate-rich biofilms contributing to antibiotic resistance, enabling their persistence in unfavourable environments. The cell walls of plants consist of a variety of complex glycans and the most abundant molecule in the biosphere is cellulose, a homopolymer of β1,4 linked d-glucose, forming the primary constituent of secondary cell walls and providing plants much of their structural rigidity. Additionally, plant primary cell walls contain a complex variety of hemicellulose, pectins, rhamnogalactans and others^2,3^ whose coordinated modification enable growth and provide both support and flexibility.

Mankind has long isolated and re-purposed naturally occurring complex glycans for ‘non-natural’ uses of technological and societal value. Currently, cellulose is employed in filtration and dialysis membranes; the hemicellulose, xyloglucan, is used in industrial surfactants; the HS-like glycan, heparin, whose biological role is still not fully understood, is exploited as a major anticoagulant drug and the polysaccharides xanthan gum, carrageenan and gum arabic (from bacterial, algal and plant sources, respectively) find applications that include use as thickeners for food. Purification of such polysaccharides can involve significant effort and natural batch-to-batch variation also presents challenges to product consistency, thus, approaches that enable biotechnologically relevant complex carbohydrates to be accessed which are both economically viable and reproducible, would be desirable.

Despite the diverse and essential roles that complex carbohydrates serve in nature, and their considerable industrial value through deliberate re-purposing, the sequence and conformational landscape of complex carbohydrates that are explored by nature is, in fact, very small in comparison to that which is hypothetically available. Complex carbohydrates comprise more individual building blocks than proteins, their sugar building blocks may be d- or l-configured, possess either α or β glycosidic bonds, involve 5- or 6-membered (furanose and pyranose) rings, and can be attached through several potential linkage positions to their neighbours in both a linear or branched manner. Even ignoring other potential modifications, such as phosphorylation, sulfation and acetylation, this complexity provides this class of molecule much more structural variety than is available to proteins. Just as in proteins, however, structure is related intimately to the function of the molecule, and can be finely adjusted through subtle structural changes.

Accessing more of this unexplored sequence and conformational carbohydrate space would provide new polysaccharides (NPs) that do not exist in nature, with unique chemical and physical properties of potential value. Documented approaches have been reported for the synthesis of polysaccharides starting from the reaction of suitably protected intermediate glycosyl donor and acceptor molecules,^4^ as well as reports of pseudo-polysaccharides with hybrid or unnatural linkages (*e.g.* amines, orthoesters and carbamates),^5^ and also those that employ ring opening polymerisation.^6^ The use of protected donors and acceptors, however, can be expensive and time consuming, and is usually restricted to the synthesis of oligosaccharides.

**Fig. 1:**
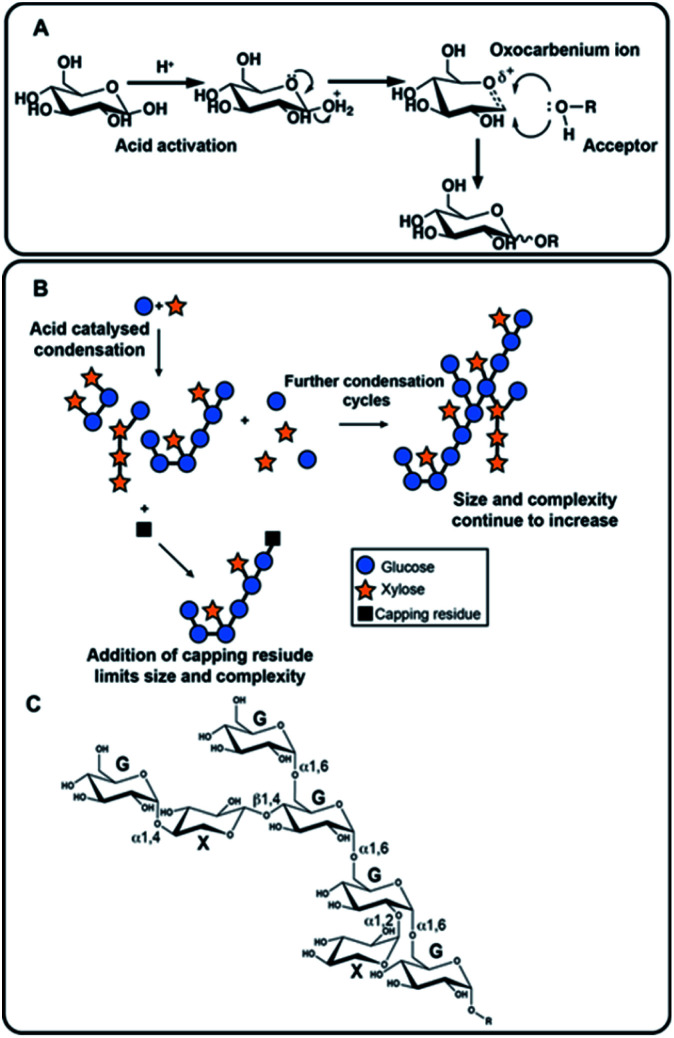
The mechanism of acid catalyzed synthesis of new polysaccharides and illustrative examples of the products which can be generated by the process. (A) Mechanism of acid catalyzed glycosylation (shown for d-glucose in the pyranose ring form) that allows addition of a sugar unit (HO–R, the acceptor) to the reducing end (of the donor). Nucleophilic attack is possible from either face and the thermodynamically most stable will predominate. (B) The product is then available to act subsequently as both acceptor and as donor through several hydroxyl groups, tending to lead to complex branched products. Many adaptations are available, for example, here the use of a capping residue is shown, which may serve to limit polymerization, or to introduce a labelled moiety for instance. (C) An example of a hypothetical structure formed from condensation of d-glucose (G) and d-xylose (X).

The approach adopted here to generate NPs is the acid-catalysed condensation of simple sugars. This reaction has a long history and is based on Emil Fischer's 1895 acid catalysed formation of methyl glycosides from monosaccharides and methanol,^7^ then polysaccharides,^8^ and which was later adapted to employ catalysts such as phosphoric acid.^8,9^ These approaches have subsequently been adapted further^10,11^ and here, we apply microwave heating to drive the condensation of simple, unprotected sugars to generate a series of exemplar NPs (Fig. 1), catalyzed by environmentally benign organic acids, such as citric acid, and explore their potential in a range of biological assays. This approach avoids the environmentally damaging metal salts, such as tin or zinc chlorides and silver triflate of some conventional Lewis acid catalysed glycosylations, and can generate NPs of several kDa within minutes. The NPs formed can then be purified easily by dialysis or gel permeation chromatography before processing for analysis or downstream applications. The approach generates polysaccharides following the most favourable thermodynamic route and foregoes formal control over regio- and stereoselectivity. The properties of the resulting NPs can still be influenced to some extent through the choice of the initial reactants and their complexity can be increased by conducting repeated rounds of synthesis introducing varied reactants. It should be noted, however, that the primary goal in this initial exploration is to create *de novo* NPs which do not exist in nature and whose biological activities, or biotechnological applications, are initially unknown, thus, the generation of pre-determined structures is not a formal aim.

Furthermore, in addition to the *de novo* approach, a functionalisation approach can be adopted. Existing polysaccharides can be modified using this second route to alter their properties. The starting material for functionalisation could vary from existing materials with the aim of modifying their properties, to waste materials such as those sourced from biotechnological processes (*e.g.* biofuel reactors) with the aim of generating products with useful activities and, hence, added value.

We describe the application of microwave induced acid-catalysed condensation to generate NPs through both routes. We demonstrate that, without further optimization, *de novo* polysaccharides exhibit diverse biological activities that include influencing bacterial biofilm formation, acting as mimetics of tuberculosis arabinans and of epitopes on the cell surface of parasites. Illustrating the functionalisation route, chondroitin sulfate that has been fucosylated and chemically sulfated, inhibits cell growth in the presence of heparin-dependent growth factors. These data demonstrate that NPs are not biologically inert and can interact with biological systems in new ways. New polysaccharides can be produced quickly, simply, and the method is amenable to operating at scale. Thus, NPs have enormous potential to drive an expansion in the use of complex carbohydrates in both biological and industrial settings, utilising a simple, rapid and environmentally-friendly synthetic process.

## 2. Results

We describe below five examples of the generation of novel polysaccharides (NPs) using the present approach without further optimisation. Four examples employ the *de novo* synthetic route, starting from monosaccharide building blocks to construct non-natural glycans, thereby exploring novel chemical space. The fifth and final example involves functionalisation of the naturally occurring glycan, chondroitin sulfate-A, with l-fucose followed by chemical sulfation, to introduce new biological functionality into an existing polysaccharide.

### Generation of polysaccharides able to influence biofilm formation by *Pseudomonas aeruginosa*

*Pseudomonas aeruginosa* (*P. aeruginosa*), an opportunistic pathogen, is responsible for infection in vulnerable individuals and is particularly common in clinical settings. For instance, *P. aeruginosa* contamination of medical devices is a widespread problem, and can cause pneumonia in immunocompromised patients, as well as persistent infections of the lungs of cystic fibrosis sufferers. *Pseudomonas aeruginosa* exhibits extensive drug resistance and is also able to form biofilms, presenting additional resistance to drug treatment and cleaning procedures. Biofilms are complex structures containing polysaccharides, proteins, nucleic acids and lipids, that are able to adhere to the surfaces of tissues and implanted devices and thereby form an environment that protects the bacteria from both antibiotics and the immune system of the host, as well as providing a site in which other bacteria may become resident.^12^

**Fig. 2:**
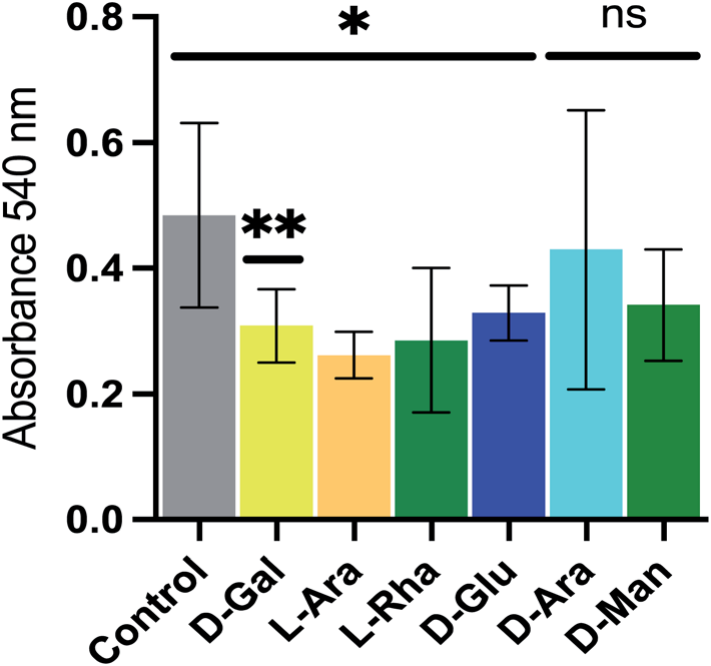
The ability of novel polysaccharides to alter biofilm formation. New polysaccharides formed by condensation of d-galactose, l-arabinose, l-rhamnose or d-glucose inhibited biofilm formation at 100 μg mL^−1^. ns indicates no significant difference.

To investigate the use of NPs as novel reagents in disrupting biofilm formation we employed the *de novo* synthetic route to generate several NPs, each using a simple sugar (d-galactose, l-arabinose, l-rhamnose, d-glucose, d-arabinose and d-mannose respectively) as the building block catalyzed by citric acid and formed complex, branched polymers typically of ∼5 kDa. These were added at a concentration of 100 μg mL^−1^ to bacterial cell cultures of *P. aeruginosa*, and the effect on biofilm formation monitored using a crystal violet-based method of biofilm detection.^13^ The effects on biofilm formation of *P. aeruginosa* by the NPs was varied; the d-galactose NP caused a *ca.* 36% reduction in biofilm formation, while others reduced biofilm formation less dramatically (l-arabinose, l-rhamnose and d-glucose NPs) or not at all (d-arabinose and d-mannose NPs) (Fig. 2). The mechanisms behind the action of these NPs, which have not been optimized, are unknown, but the potential utility is clear.

### Poly d-arabinose NPs are recognised by anti-lipoarabinomannan (LAM) polysaccharide antibodies

The review by Peltier *et al.*^14^ summarizes over fifty polysaccharides from microorganisms that contain both pyranose and furanose ring forms of hexoses. Of these, about one third were recognized as playing a role in pathogenic processes and included major disease-causing genera such as *Mycobacterium*, *Shigella* and *Trypanosoma*. During the mutarotation process undergone by free monosaccharides in aqueous solution, five-membered furanose rings form more quickly than six-membered pyranose rings but, unlike pyranoses, furanose ring forms do not favour significantly the formation of particular anomeric configurations, suggesting that complex NP structures are likely to result during the condensation process. Some of the structures formed are likely to resemble naturally occurring sequences, while others are expected to be new.

**Fig. 3:**
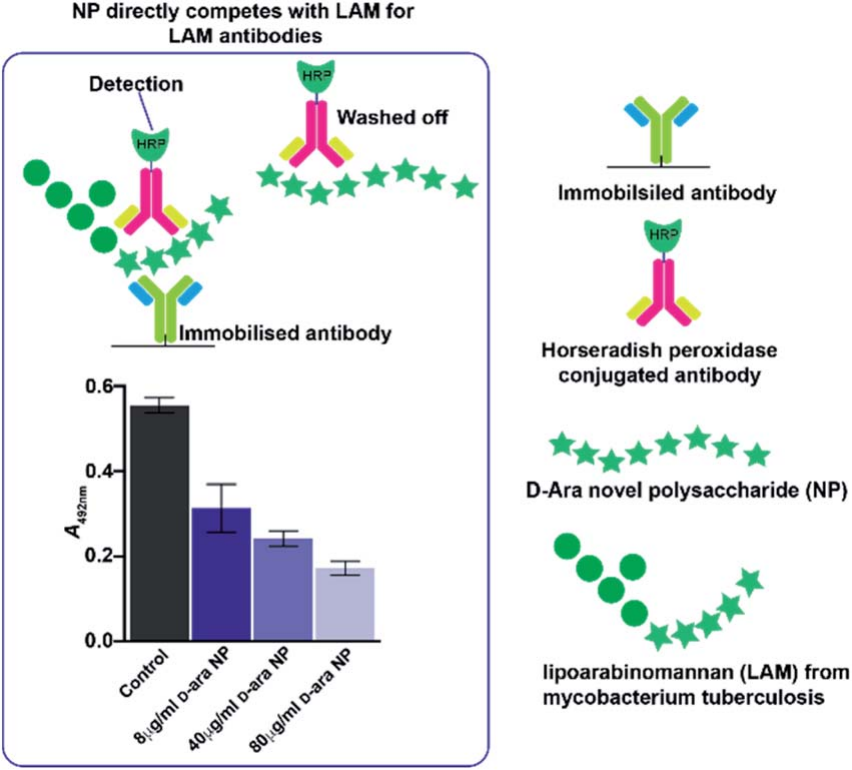
Novel polysaccharides generated from d-arabinose can act as mimics of lipoarabinomannan from *Mycobacterium tuberculosis*. The arabinose NP is able to compete (applied at 8, 40 and 80 μg mL) for binding to the anti-LAM primary antibody.

**Fig. 4:**
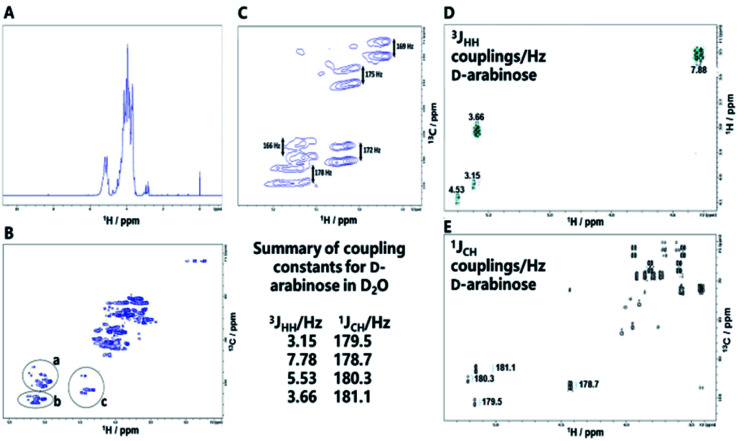
(A) ^1^H NMR spectrum of the NP formed by condensation of d-arabinose. (B) Decoupled HSQC NMR spectrum of the NP of d-arabinose revealing its complex structure. Inset: anomeric regions containing signals provisionally assigned to; ((a) β d-arabinopyranose and terminal β d-arabinofuranose. (b) −2 linked α d-arabinofuranose, −3,5 linked d-arabinofuranose, −2,3,5 linked d-arabinofuranose and terminal d-arabinofuranose. (c) α d-arabinopyranose^33,34^). (C) Coupled HSQC NMR spectrum of anomeric region for d-arabinose NP in water (D_2_O) showing ^1^*J*_CH_ couplings between H-1 and C-1. (D) Coupled COSY NMR spectrum of anomeric region for d-arabinose in D_2_O showing ^3^*J*_HH_ couplings between H-1 and H-2. (E) Coupled HSQC NMR spectrum of anomeric region for d-arabinose showing ^1^*J*_CH_ couplings between C-1 and H-1. The inset summarizes the anomeric coupling constants for d-arabinose.

In *Mycobacterium tuberculosis*, two polysaccharides with immune-modulatory properties predominate; lipoarabinomannan (LAM) and arabinogalactan (AG), which both contain furanose ring forms. In the branched LAM polysaccharide, β (1-6) and β (1-5) linked d-galactofuranose, as well as β d-arabinofuranose, have been identified.^15–17^ Structures comprising these furanose ring forms are of particular interest, since they are not found in mammals and thus they could act, or be modified to act, as potential enzyme inhibitors against mycobacterial transferases or, as a source of antigenic structures to prime the immune system. The LAM structures include terminal sections that represent epitopes responsible for the antibody response evident commonly in mycobacterial infections.^17^ The availability of an anti-LAM antibody, used as a diagnostic for the disease, offers the possibility of identifying epitopes based on d-arabinose rapidly, for future exploitation, using the *de novo* synthetic route. To this end, an NP was produced from d-arabinose and the ability of the NP to compete with binding of the antibody to the positive control, was demonstrated (Fig. 3). This d-arabinose NP was also subjected to analysis by NMR which confirmed its complex structure, comprising anomeric linkages of both α and β pyranose and furanose ring forms (Fig. 4).

NMR revealed signals for both free primary hydroxyl groups and those linked by glycosidic bonds, evident in the HSQC spectrum with ^13^C chemical shifts around 64 ppm and 69–70 ppm respectively.^18^ The analysis highlights the complexity of the NP products formed, even from a single starting sugar, in this case revealing both α and β d-arabinose in both furanose and pyranose forms.^19^ Measurement of one (^1^*J*_CH_) and three bond (^3^*J*_HH_) NMR coupling constants can provide information regarding anomeric configuration and conformation; measurements of free d-arabinose, provided ^3^*J*_H1H2_ and ^1^*J*_C1H1_ values for reference of 3.15/179.5 Hz and 4.53/180.3 Hz for d-arabinofuranose and 3.66/181.2 Hz and 7.78/178.7 Hz for α and β d-arabinopyranose anomeric forms, respectively. The ^1^*J*_CH_ couplings for the pyranose forms in the d-arabinose NP (Fig. 4C) corresponded relatively closely to those of free d-arabinose (Fig. 4E), consistent with ^4^C_1_ chair forms but, those of the furanose forms varied, most likely indicating a conformation for these within the polymer that differs from the expected ^2^T_3_ form of free d-arabinofuranose in solution.^18^ Arabinan structures extracted from *Mycobacterium* spp.^15–17^ lipoarabinomannan (LAM) that are considered responsible for the immunogenic response, contain a range of linkages and configurations, including β d-arabinofuranose (1-2), α d-arabinofuranose, -3 and -5 linked α d-arabinofuranose residues, as well as terminal d-arabinofuranoside units;^17^ signals consistent with some of these structures were also evident in the NMR spectra of the d-arabinose NP.

### Generation of polysaccharides recognized by a lectin that binds α d-galactose structures that are characteristic of the surfaces of trypanosomes

Oligo- and polysaccharide structures terminating with α d-galactose are characteristic glycan structures on the cell surfaces of trypanosomes and, since α d-gal linked structures do not occur in humans, they have been proposed as both diagnostic tools and as components of possible vaccines.^20^ Indeed, a conjugate of an α (1-6) d-galactose disaccharide and BSA was shown to be protective against *Leishmania major* infection.^21^ The α-d-galactose epitope has also provided an antigenic obstacle to xenotransplantation, since it is present in most other mammalian species. Following bites by ticks, α (1-3) d-galactose disaccharides can also be the source of acquired allergy to meat in the diet. It has been suggested that the loss of α-d-galactose biosynthetic capability in humans and apes, which occurred 28 million years ago, bestowed a selective advantage by providing immunogenic protection against a number of pathogens (discussed in ref. 22).

**Fig. 5:**
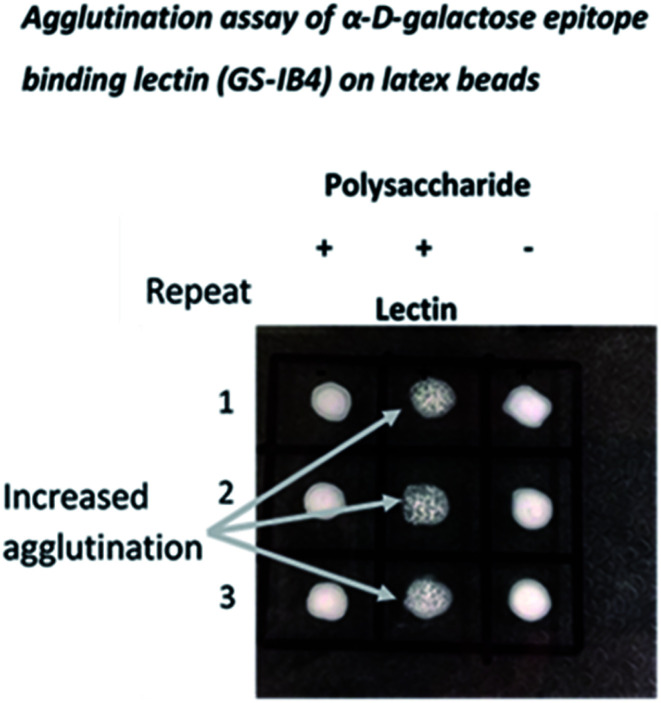
Agglutination assay employing an NP formed by condensation of d-galactose. The NP formed by condensation of d-galactose is recognized by a lectin (isolectin GS-1B4 from *Griffonia simplicifolia*) specific for α d-galactose epitopes such as those on the surface of Trypanosomes. The lectin was coated on latex beads and addition of the NP resulted in agglutination.

Here, the *de novo* synthesis route was used to generate NPs with potential utility in these applications, and the detection of α d-gal epitopes was demonstrated using a lectin from *Griffonia simplicifolia* with high affinity for α linked d-galactose units (Fig. 5).

### New polysaccharides from a biotechnologically-derived starting material, d-allulose

The approach also provides a route to generate NPs from biotechnologically-derived starting materials and can include those produced from rare or novel monosaccharides. An example is provided by the condensation of d-allulose (d-psicose), the C-3 epimer of d-fucose, which is used as a low-calorie dietary sweetener, and is found only very rarely in nature. d-Allulose is produced on a large scale by biotechnological approaches,^23^ however, and the *de novo* route generates NPs with potentially novel attributes that presumably include polysaccharide structures to which existing carbohydrate degrading enzymes have never been exposed. d-Allulose was condensed using two different organic acid catalysts, citric (p*K*_a(1)_ 3.1) and *cis*-aconitic (a dehydrated derivative of citric acid, p*K*_a(1)_ 2.8) acids. The NP produced with citric acid catalysis exhibited a molecular weight by viscosity measurements against dextran standards of *ca.* 5.3 kDa, while that of the *cis*-aconitic acid catalyst was lower, at *ca.* 4 kDa (Table 1).

**Table 1:** Molecular weight determination of d-allulose NPs formed under catalysis by citric acid and *cis*-aconitic acid by viscosity measurements relative to dextran standards

	Flow times (*t*/*t*_0_) in viscosity apparatus				
Molecular weight dextran standards/kDa	7	12	16	21.4	196
*t*/*t*_0_ (rel. to H_2_O = 1.00)	1.29	1.51	1.59	1.84	4.37
d-Allulose NP (citric acid) (SD)	1.22 (0.3)	[*M*_w_*ca.* 5.3 kDa: dp 24/25]			
d-Allulose NP (*cis*-aconitic acid) (SD)	1.17 (0.2)	[*M*_w_*ca.* 4 kDa: dp 33]			

### Generation of a fucosylated glycosaminoglycan *via* functionalisation and its subsequent sulfation enabled modulation of FGF cell signaling

**Fig. 6:**
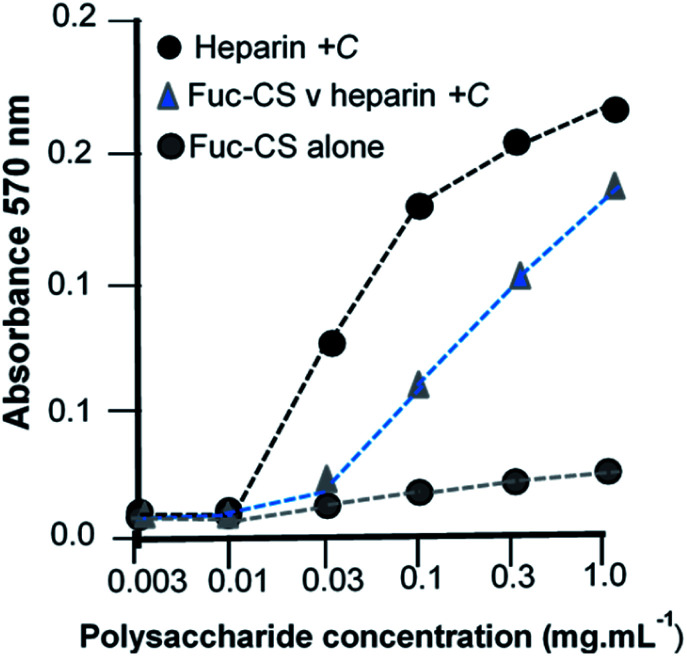
Functionalised chondroitin sulfate is a competitive inhibitor of fibroblast growth factor signaling. The functionalized chondroitin sulfate (CS) NP was titrated against the positive control, heparin. Heparin serves as mimic of heparan sulfate, the endogenous and obligatory co-receptor of FGF signaling, and supports signaling between FGF1 and FGFR1c in BaF cells that have been modified to lack the ability to synthesise heparan sulfate. Chondroitin sulfate alone does not signal through FGF/FGFR1c.

Finally, we illustrate the generation of a NP made by the functionalisation route. As an example, l-fucose was reacted with an existing polysaccharide, the glycosaminoglycan, chondroitin-4-sulfate (chondroitin sulfate-A), followed by an *O*-sulfation step and the sulfated product was tested for its ability to compete against heparin in a cell-based assay of signalling. In this assay, heparin acts as a proxy for the naturally occurring heparan sulfate. The BaF cell line is devoid of endogenous heparan sulfate, which is the obligatory co-receptor in FGF-FGFR tyrosine kinase driven cell signalling,^24^ and cells were transfected with a single, selected FGFR (FGFR1c), then cultured in the presence of a chosen FGF (FGF-1) together with heparin which served as the positive control. The test NP, chemically sulfated fucosylated-CS-A, was then co-incubated under these conditions as a potential inhibitor and its ability to compete the cell proliferation capability of the FGF-heparin combination was determined (Fig. 6).

The FGF-driven coordination of wound and nerve repair as well as developmental processes, are all areas of extensive research. Derivatisation of existing materials such as GAGs, many of which have already found application in practical devices, offer a direct means of further tailoring their properties.

## 3. Discussion

The number of hypothetical sequence arrangements of the amino acids that comprise a typical protein is vast, but it is not appreciated so widely that this complexity is dwarfed by the possible variation provided by carbohydrates of much shorter lengths. Complex carbohydrates fulfil numerous, varied and essential roles throughout nature, indeed, the emergence of eukaryotic life closely tracks the emergence and complexity of sulfated glycans at the cell surface.^25^ Despite this, to facilitate life, nature has only tapped into a small subset of the plethora of complex glycans that potentially could be generated. Furthermore, mankind utilises this limited source of naturally occurring glycans for a wide range of uses for which they did not evolve. This leads to the tantalizing speculation as to what could be achieved were the vast chemical and conformational space of complex carbohydrates to be explored more comprehensively.

Among the immediately applicable uses would be the generation of NPs to replace existing polysaccharides for which purification and natural variation issues arise. For example, xyloglucan, which can be used in surfactants^26^ is known to vary from batch to batch depending on the source,^27^ necessitating regular optimisation of processes. It would be feasible to generate NPs with similar properties from either the same sugar building blocks as xyloglucan, or from others bearing no obvious relation, but that yielded a polymer exhibiting similar or enhanced properties. The use of polysaccharides as food thickeners is another area in which NPs, could readily provide replacement polysaccharides and thereby avoid time-consuming methods of purification, whether the polysaccharides in current use originate from marine, terrestrial, or bacterial sources. For example, the extraction of xylan from terrestrial plants is known to alter the structure of the original material under highly alkaline conditions, by removing acetyl and ferulic acid groups.^28,29^ For some applications, these groups need to be replaced chemically. Again, by beginning to explore carbohydrate chemical space through microwave assisted acid-catalysed condensation, it may be possible to generate substitutes for xylan with similar properties in a more environmentally responsible manner.

The generation of NPs that contain entirely new structures, such as those from d-allulose raises a further interesting possibility. This is to test both the degree to which extant enzymes are specific and to explore the possibility of evolving new enzymes experimentally that are capable of degrading such new structures, for example, using bacterial polysaccharide degrading enzymes. Two types of potential substrate can be envisaged. The first comprises NPs made from naturally occurring monosaccharides whose polysaccharides contain both naturally occurring and alien polymerised structures, while the second employs an unnatural or very rare sugar, such as d-allulose, to generate polysaccharides that have never been encountered in nature. This would provide an experimental regime under which the evolution of carbohydrate active enzymes could be monitored with defined materials, providing insights into how new carbohydrate active enzymes evolve.

The ability to inhibit biofilm formation could provide a role for optimised NPs as a protective coating on surfaces and devices but, those able to support biofilm formation could be used in a sacrificial role or perhaps even to promote biofilm formation at a location of choice should this be desirable. The ability of *P. aeruginosa* to evolve to the effects of a NP would also be much reduced compared to antibiotics, as the genetic tools to degrade these NPs do not exist in nature. Should resistance begin to develop, the ability to either generate new NPs or functionalise existing ones is a rapid process.

The biological activities of the NPs generated in this work serve as a proof of principle and required no optimisation to demonstrate their biological functions; there is, therefore, no question whether newly generated NPs will have biological and industrial applications but, rather, what those will (or could) be. Several general routes by which the structures of NPs could be influenced include the timed addition of reactants with contrasting properties, for example, adding a charged sugar to an already formed branched but neutral scaffold, or the deliberate manipulation of equilibrium composition, exploiting the relatively rapid equilibration of furanose and pyranose forms or their slower anomerisation, or manipulation through pH, and the use of cations. Some control over the degree of branching, the use of conventionally protected sugars can also be envisaged to provide initial scaffolds for polymerisation. This would enable, for example, NPs with some pre-designed characteristics to be synthesized.

Despite its origin in a reaction that harks back to the early days of carbohydrate chemistry, microwave assisted acid catalysed condensation offers a rapid, facile method for beginning to explore the enormous untapped potential of carbohydrate sequence and conformational space. Starting from widely-available simple sugars and employing environmentally benign catalysis, it promises new materials, some of which will be able to replace current chemicals in products such as cosmetics, as well as generating new materials with completely novel functionality.

## 4. Materials and methods

### General synthetic procedure

A chosen reducing sugar (monosaccharide hexose (500 mg), or pentose (417 mg), 2.78 mmol) was dissolved in an aqueous solution of the organic acid catalyst (citric (50 mg) or *cis*-aconitic acid (45 mg (0.26 mmol)) in 2.5 mL water) in a 10 mL conical flask plugged with glass wool, and was degassed using nitrogen (5 min). The mixture was microwaved (medium–high power (4 min) followed by high power (1 additional minute)) in an atmosphere of nitrogen. The thick syrup, often exhibiting colouration ranging from very pale yellow to dark brown, was then allowed to cool and was re-dissolved in water (10 mL), dialysed against water (2 L, 3.5 kDa cut-off) and dried by rotary evaporation before further analysis by NMR and other physico-chemical techniques, or use in biological assays. As an example, the NP made using this method from 417 mg of d-arabinose with citric acid catalysis, yielded 60 mg with *M*_r_ > 3500 Da (nominal cut-off of the dialysis tubing).

### Generation of fucosylated chondroitin sulfate-A and its subsequent sulfation

The GAG oligosaccharide, chondroitin sulfate-B (*M*_w_ 26 kDa) was functionalised by reaction of CS-A (200 mg) and l-fucose (30 mg), citric acid (40 mg) in water (2.5 mL) over 4 min in a commercial microwave oven to generate fucosylated CS (Fuc-CS) with *M*_w_ 34 kDa (determined by viscosity measurements relative to dextran standards). Subsequent *O*-sulfation was achieved by reacting the fucosylated product (52 mg) with 3 eq. pyr. SO_3_ complex in DMF (3 mL) overnight at room temperature (16 °C), followed by neutralisation to pH 8 (0.1 N NaOH), precipitation in cold ethanol and dialysis (3.5 kDa cut-off v H_2_O) to recover the product and the dialysate was dried for further use.

### Assay of biofilm formation by *Pseudomonas aeruginosa*

*Pseudomonas aeruginosa* was cultured under standard conditions.^30^ Novel polysaccharides, made from the condensation of l-arabinose, l-rhamnose (see General synthetic procedure above) and Fuc-CS were formed as described above purified by dialysis (>3.5 kDa (dialysis)) and assayed (100 μg mL^−1^) for their influence on biofilm formation (*Pseudomonas aeruginosa*), in a 96-well assay that detects biofilm formation through staining with crystal violet.^13^

### Inhibition assay of FGF signaling in BaF cells

BaF3 cells (murine lymphocytes) were transfected with the FGF receptor (FGFR1c) and maintained in RPMI-1640 supplemented with 10% fetal calf serum, 2 mM glutamine, 100 U nL^−1^ pen G, 1 nM FGF1, 50 μg mL^−1^ streptomycin sulfate and 2 ng mL^−1^ IL-3. Assays for cell function were as described.^19^ Chondroitin sulfate alone does not support signalling through FGF1/FGFR1c. Positive control experiments included addition of porcine mucosal heparin (3–1000 μg mL^−1^) and inhibition involved addition of the test NP, sulfated l-fucosyl chondroitin sulfate-A (*M*_w_ 34 kDa, 3–1000 μg mL^−1^) in the presence of heparin at 1000 μg mL^−1^.

### Inhibition of anti-LAM antibody binding

Assays were conducted using a commercial lipoarabinomannan ELISA kit (Aviva Biosystems Biology, San Diego, USA) in two formats – one in which direct antibody binding to an NP (made by condensation of d-arabinose and previously coated onto an ELISA plate) was measured (results not shown). The d-arabinose NP was coated at 100 μL in PBS per well (72 h, room temp.) followed by washing and blocking (4% BSA in PBS) and antibody probing (1/1000 dilution in PBS from 1 mg mL^−1^ in 4% BSA in PBS) incubation (1 h), washing and detection by probing with HRP conjugated secondary antibody and subsequent detection of tetramethylbenzidine at 450 nm. The second assay was a competition ELISA, in which the d-arabinose NP served as a potential competitor (applied at 8, 40 and 80 μL mL^−1^) for the binding of the anti-LAM antibody to the positive control (manufacturer's instructions).

### Agglutination assay using a lectin from *Griffonia simplicifolia* for α-d-galactose epitopes

Lectin-coated latex beads (Sigma, UK, polystyrene beads (0.8 μm, 0.5 mL suspension)) were prepared by incubating (overnight, 4 °C) with a solution of a lectin able to bind α d-galactose units (isolectin GS-IB4 from *Griffonia simplicifolia*) using 100 μL of a 1 mg mL^−1^ (PBS) stock solution (Invitrogen, UK) followed by extensive washing in PBS. Comparisons of agglutination were made by addition of a small volume (10 μL) of a suspension of the beads to a solution of the test NP (10 μL, 0.1 mg mL^−1^) on a glass slide.

### NMR characterisation of new polysaccharides

NMR experiments were performed on products (20 mg) in 700 μL of D_2_O at 343 K using a 600 MHz Bruker Avance II+ spectrometer fitted with a TCI CryoProbe and chemical shift values are quoted relative to DSS (0 ppm). In addition to 1-dimensional (^1^H and ^13^C) spectra, heteronuclear (HSQC and HMBC) 2-dimensional spectra, and de-coupled HSQC, coupled HSQC and COSY spectra were also recorded on a sample of d-arabinose (5 mg mL^−1^) to measure ^1^*J*_CH_ and ^3^*J*_HH_ coupling constants respectively. Spectra were processed using Bruker TopSpin and ^1^H/^13^C HSQC integration was performed using the INFOS spectrum fitting software.^31^ Proton spectrum integration was performed using Bruker TopSpin software.

### Measurement of molecular mass by viscosity

Viscosity measurements offer one way to estimate the molecular mass of NPs. This can be done by measuring the flow rates of the solvent (*t*_0_) and a series test solutions (*t*) of polysaccharides of varying, but known, molecular weights at a given concentration (5 mg mL^−1^). Plotting the relative viscosity, *η*_r_ (=*t*/*t*_0_), against concentration, *c* (g mL^−1^), the intrinsic viscosity, *η*, can be recovered using the Mark–Houwink equation (eqn (1)) and, by reference to the constants *K* and *α*, the molecular weight can be found.

1 [*η*] = *KM*^*α*^ hence, In[*η*] = *αK* In *M*

where [*η*] is the intrinsic viscosity, *M* is the molecular weight, *K* and *α* are constants for the solvent system and type of molecule, available from the literature. Literature values for the Mark–Houwink constants, *K* and *α*, for dextran in water at 20 °C (molecular weight range 70 kDa to 2 MDa)^32^ are available but, a more direct approach is to employ a series of dextrans of defined molecular weight and to construct a calibration curve to convert the experimental measurement of *t*/*t*_0_ to molecular weight (*M*_w_). Dextran standards of *M*_w_; 7, 12, 16, 21.4 and 196 kDa (5% w/v solutions in water; Pharmacosmos, Denmark) were employed for this purpose.

## Abbreviations

d-Gal: d-Galactose
l-Ara: l-Arabinose
l-Rha: l-Rhamnose
d-Glu: d-Glucose
d-Ara: d-Arabinose
d-man: d-Mannose
LAM: Lipoarabinomannan

## Conflicts of interest

The authors declare that there are no competing interests associated with the manuscript.

## Supplementary Material

RA-012-D2RA01463G-s001
