# Supplementary material for: Exploration of expanded carbohydrate chemical space to access biological activity using microwave-induced acid condensation of simple sugars
Source: RSC Adv. 2022 Apr 8;12(18):11075–83. doi: 10.1039/d2ra01463g (PMC8992359; doi:10.1039/d2ra01463g)

Electronic Supplementary Material (ESI) for RSC Advances.
This journal is © The Royal Society of Chemistry 2022

Supplementary Information

Exploration of expanded carbohydrate chemical space to access biological activity using microwave-induced acid condensation of simple sugars.

JA London, SL Taylor, I Barsukov, A Cartmell and EA Yates

HSQC NMR spectra of products of condensation of:

Figure S1. L-arabinose
Figure S2. D-galatose
Figure S3. L-rhamnose
Figure S4. D-arabinose
Figure S5. Fuc-CS with some tentative structure assignments.

Figure S6. HSQC spectra of two preparations of poly-arabinose demonstrating reproducibility between preparations.

Figure S1. HSQC Poly L-arabinose

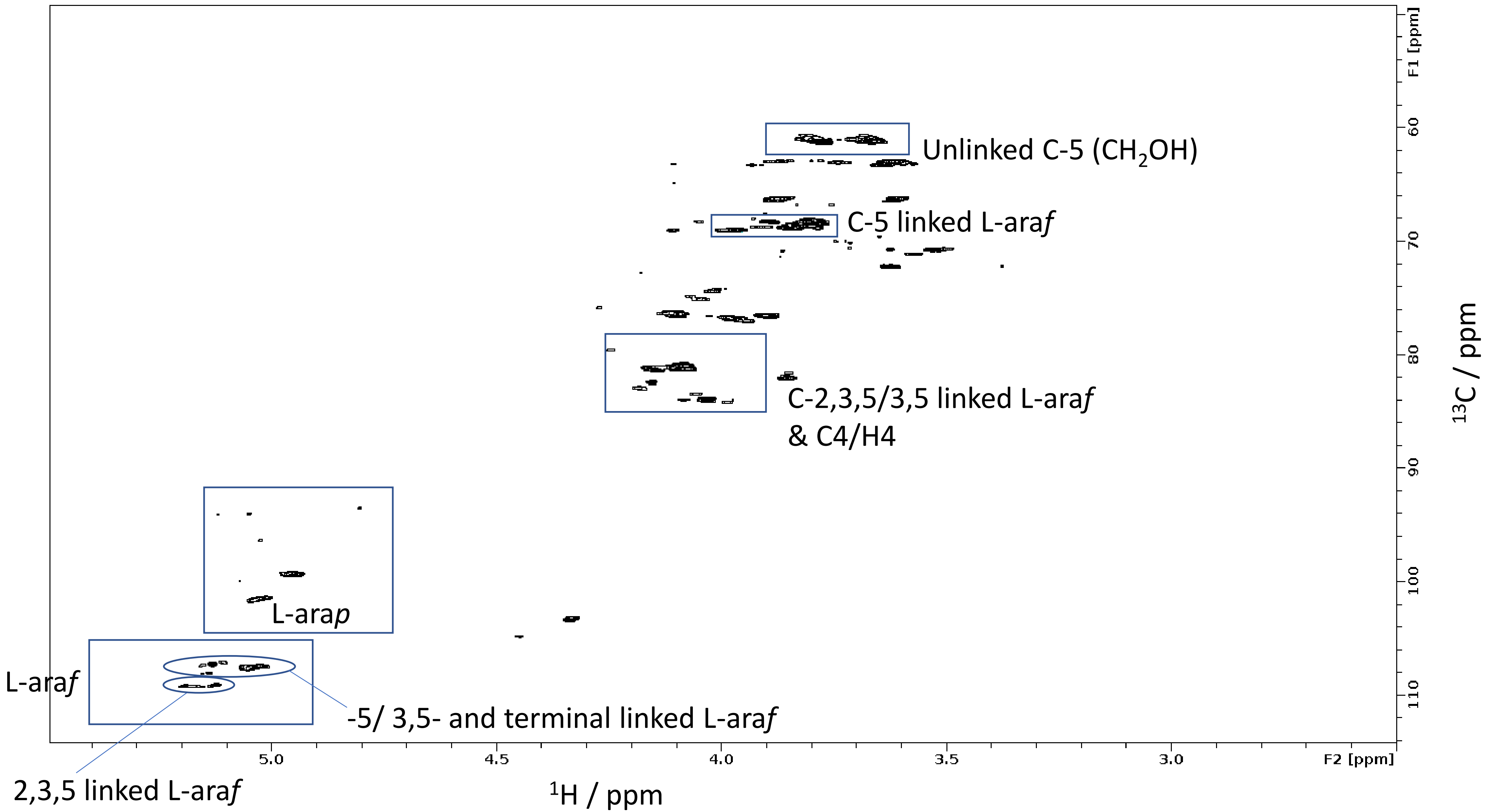

Figure S2. HSQC Poly D-galactose

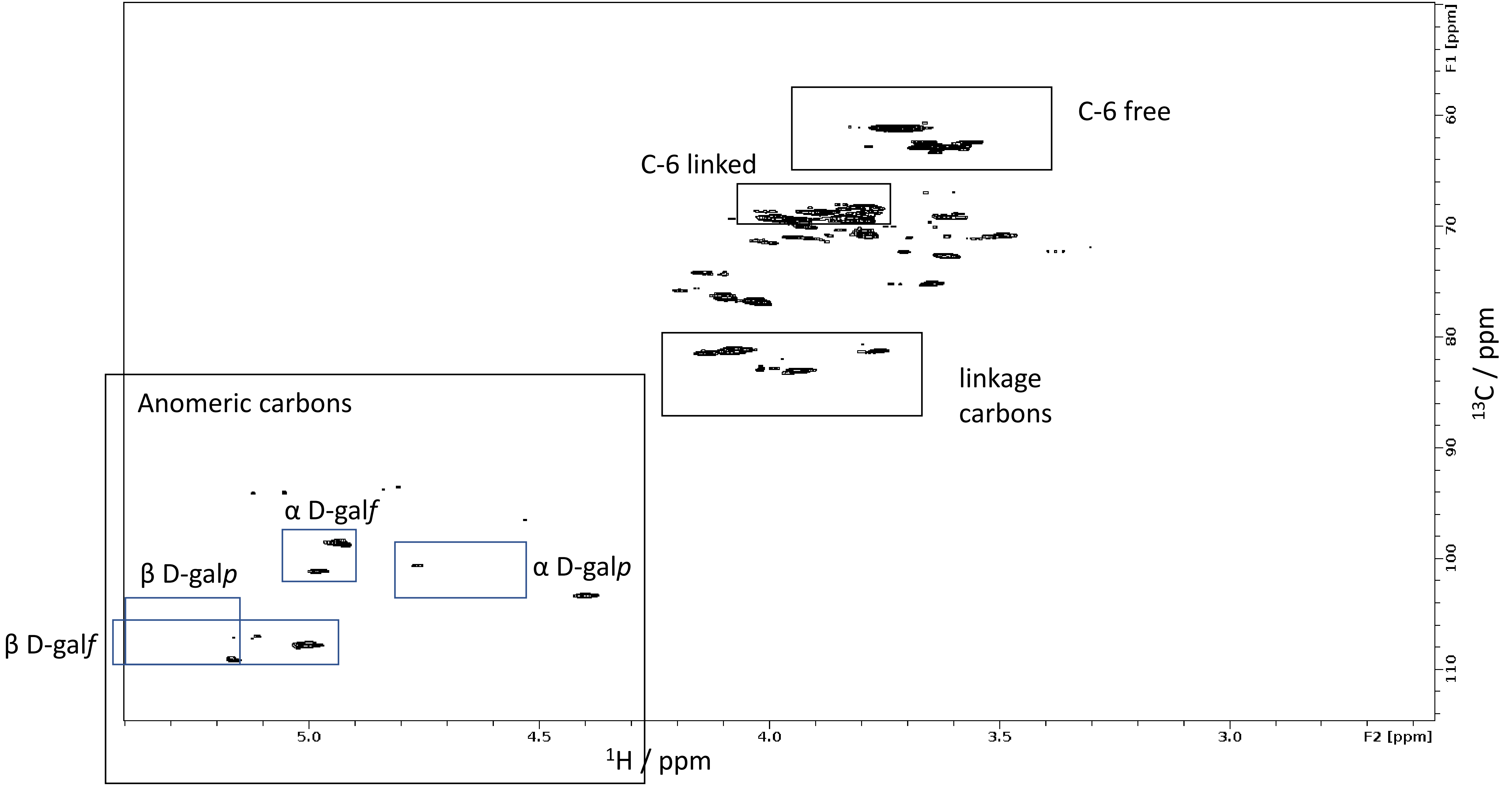

Figure S3. HSQC Poly L-rhamnose

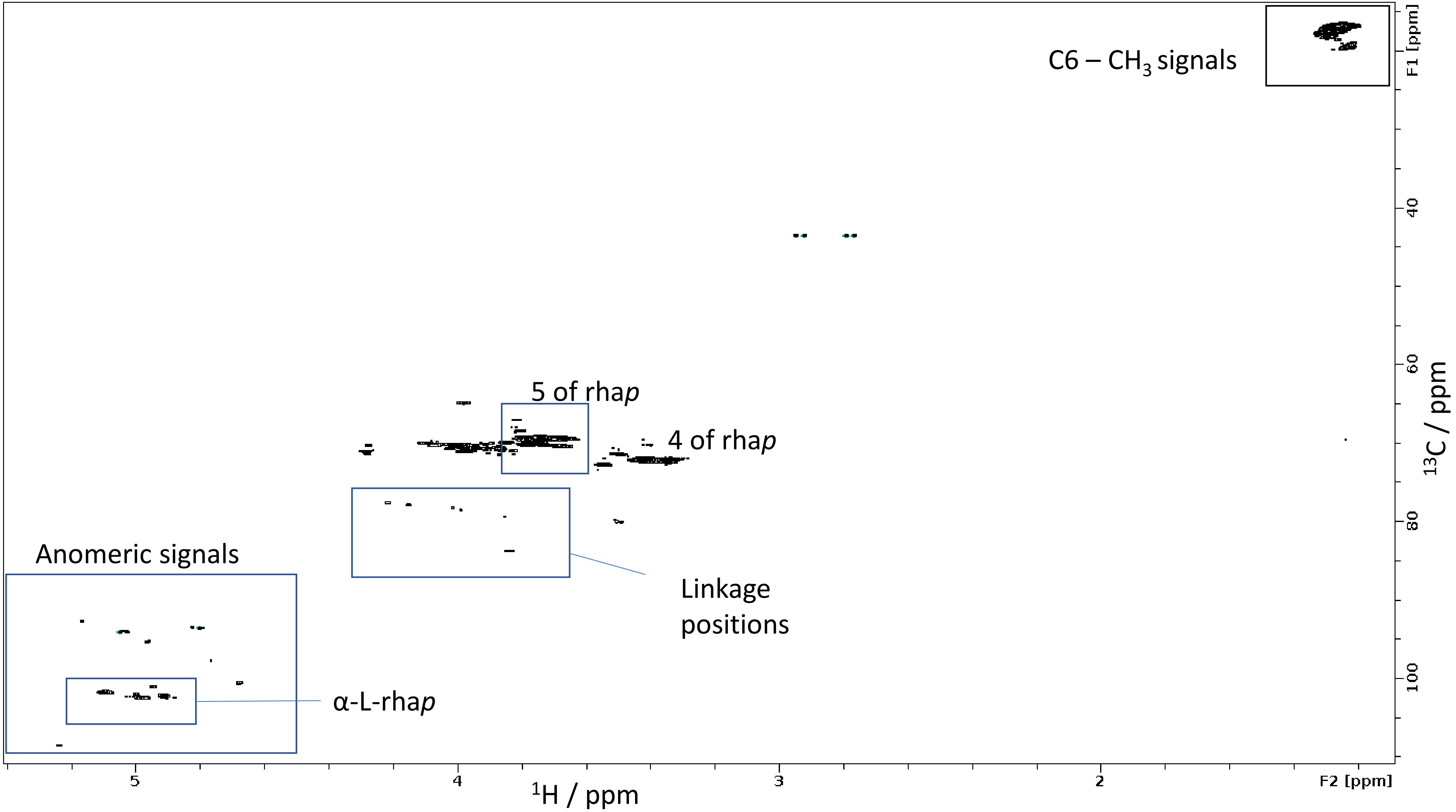

Figure S4. HSQC Poly D-arabinose

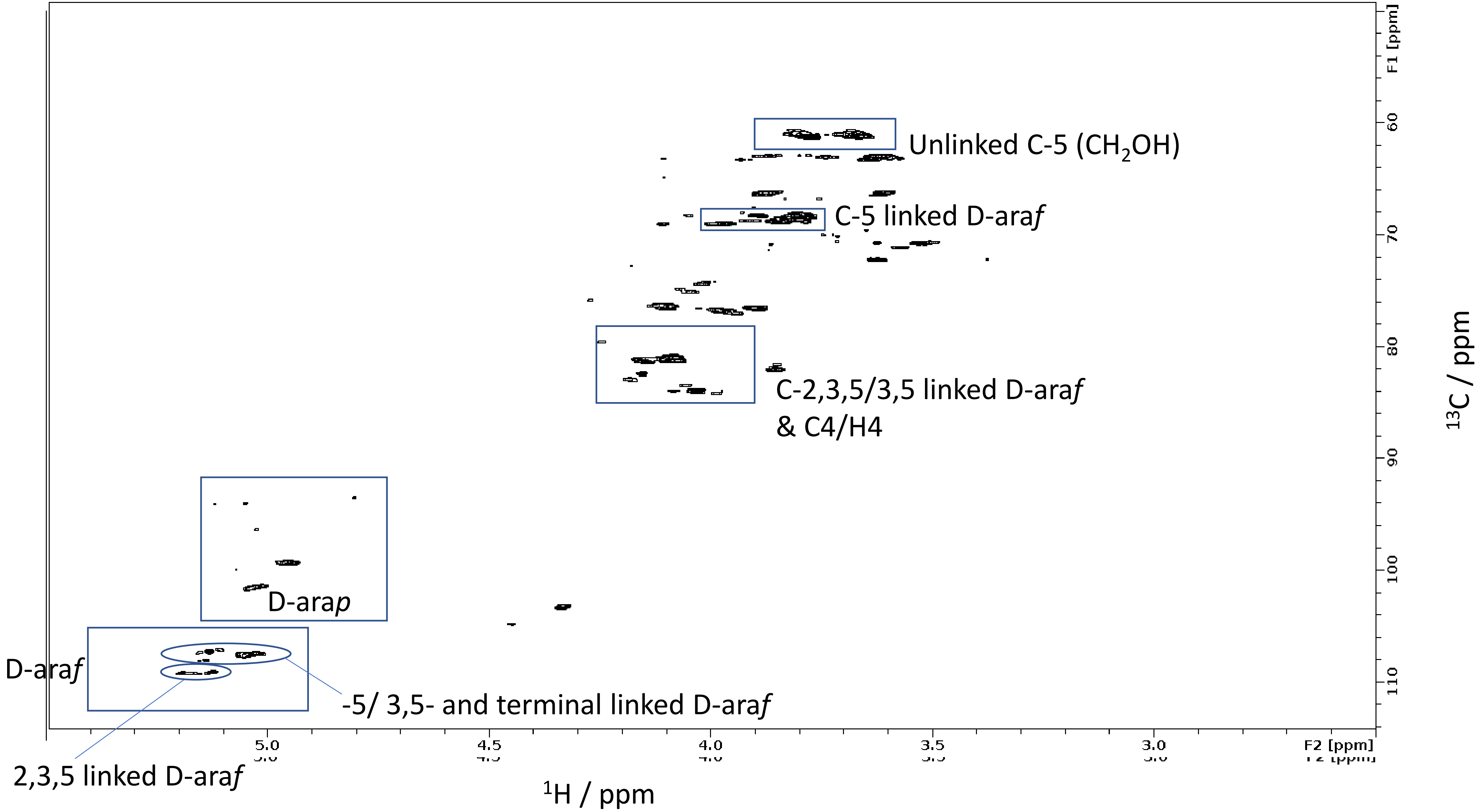

Figure S5. Fucosylated CS

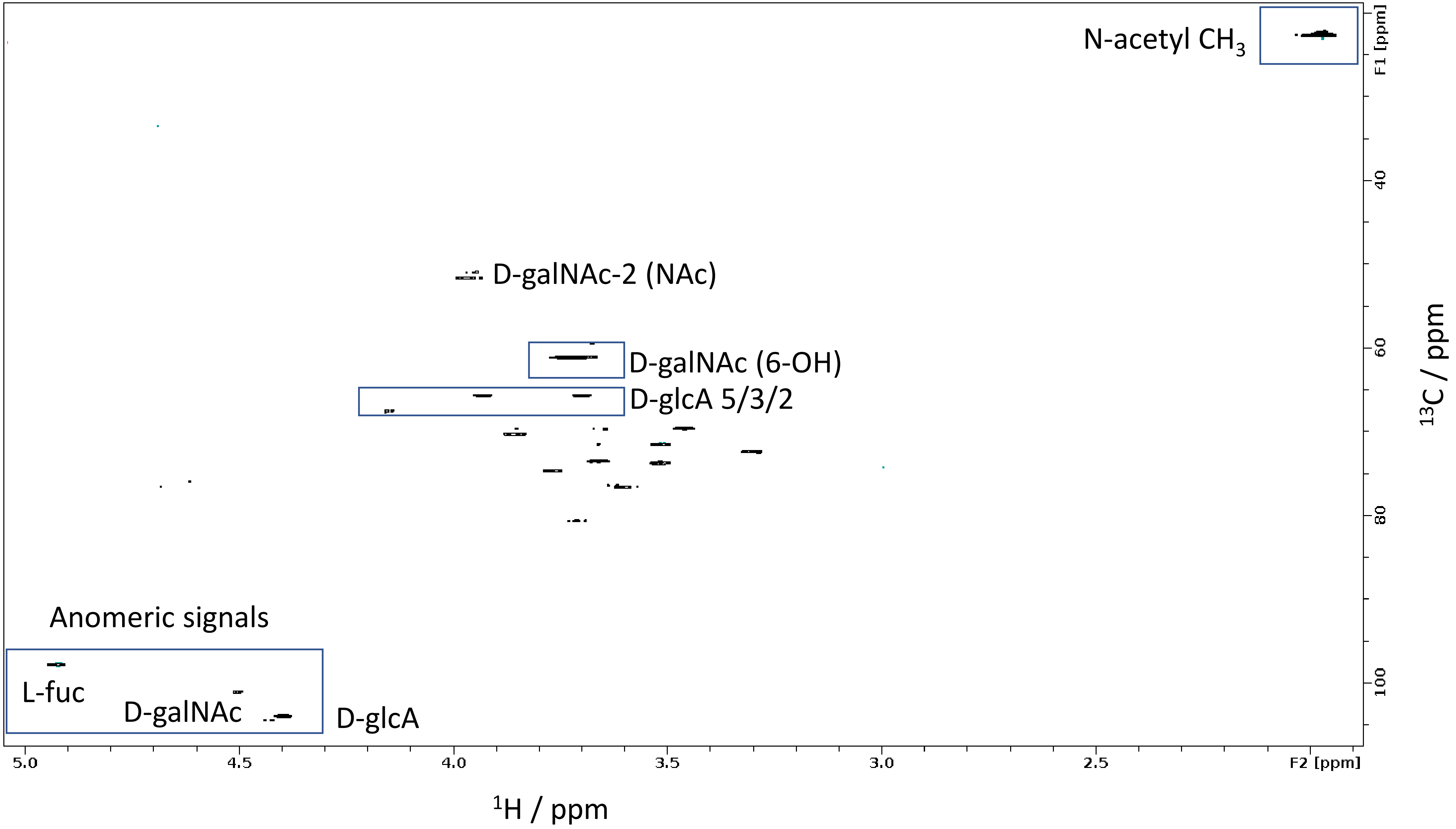

Figure S6. HSQC NMR spectra of two preparations of poly-arabinose

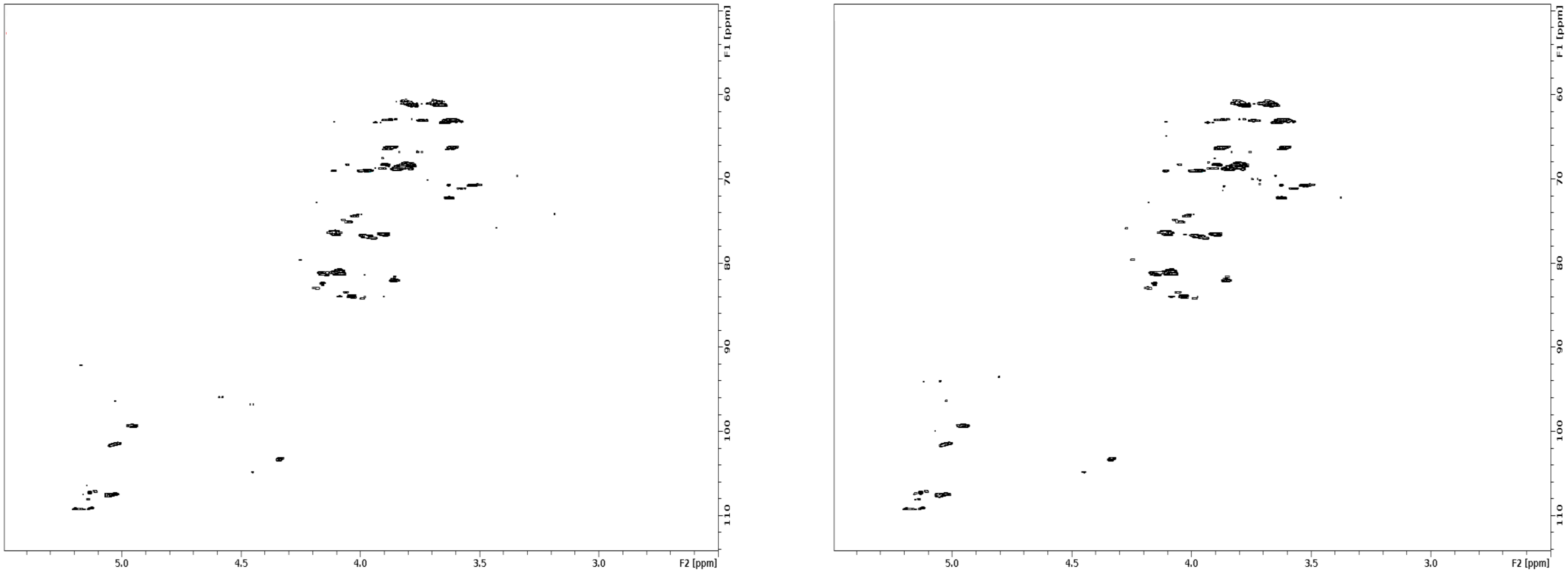

Supplement: RA-012-D2RA01463G-s001 [file RA-012-D2RA01463G-s001.pdf]
